# Exploring the Role of RGD-Recognizing Integrins in Cancer

**DOI:** 10.3390/cancers9090116

**Published:** 2017-09-04

**Authors:** Markus Nieberler, Ute Reuning, Florian Reichart, Johannes Notni, Hans-Jürgen Wester, Markus Schwaiger, Michael Weinmüller, Andreas Räder, Katja Steiger, Horst Kessler

**Affiliations:** 1 Department of Oral and Maxillofacial Surgery, University Hospital Rechts der Isar, Technische Universität München, Ismaninger Straße 22, 81679 Munich, Germany; 2 Clinical Research Unit, Department of Obstetrics & Gynecology, Technische Universität München, Ismaninger Straße 22, 81675 Munich, Germany; ute.reuning@mri.tum.de; 3 Institute for Advanced Study and Center for Integrated Protein Science (CIPSM), Department Chemie, Technische Universität München, Lichtenbergstraße 4, 85747 Garching, Germany; florian.fr.reichart@tum.de (F.R.); michael.weinmueller@tum.de (M.W.); andreas.raeder@mytum.de (A.R.); 4 Pharmaceutical Radiochemistry, Technische Universität München, Walther-Meißner-Straße 3, 85748 Garching, Germany; johannes.notni@tum.de (J.N.); h.j.wester@tum.de (H.-J.W.); 5 Department of Nuclear Medicine, Technische Universität München, Ismaninger Straße 22, 81679 Munich, Germany; markus.schwaiger@tum.de; 6 Institute of Pathology, Technische Universität München, Trogerstraße 18, 81675 Munich, Germany; katja.steiger@tum.de

**Keywords:** RGD-recognizing integrins, αvβ3, αvβ5, αvβ6, αvβ8, α5β1, α8β1, integrin adhesion, migration, apoptosis, and signaling, synthetic integrin ligands, cyclic peptide, peptidomimetics, Cilengitide, epithelial-mesenchymal transition (EMT), transforming growth factor-β (TGF-β), metastasis, angiogenesis

## Abstract

Integrins are key regulators of communication between cells and with their microenvironment. Eight members of the integrin superfamily recognize the tripeptide motif Arg-Gly-Asp (RGD) within extracelluar matrix (ECM) proteins. These integrins constitute an important subfamily and play a major role in cancer progression and metastasis via their tumor biological functions. Such transmembrane adhesion and signaling receptors are thus recognized as promising and well accessible targets for novel diagnostic and therapeutic applications for directly attacking cancer cells and their fatal microenvironment. Recently, specific small peptidic and peptidomimetic ligands as well as antibodies binding to distinct integrin subtypes have been developed and synthesized as new drug candidates for cancer treatment. Understanding the distinct functions and interplay of integrin subtypes is a prerequisite for selective intervention in integrin-mediated diseases. Integrin subtype-specific ligands labelled with radioisotopes or fluorescent molecules allows the characterization of the integrin patterns in vivo and later the medical intervention via subtype specific drugs. The coating of nanoparticles, larger proteins, or encapsulating agents by integrin ligands are being explored to guide cytotoxic reagents directly to the cancer cell surface. These ligands are currently under investigation in clinical studies for their efficacy in interference with tumor cell adhesion, migration/invasion, proliferation, signaling, and survival, opening new treatment approaches in personalized medicine.

## 1. Introduction

Cellular recognition of the interstitial neighborhood and microenvironment is required within all organisms, which depends on cell-extracellular matrix (ECM) and cell-cell communications for the correct functioning of individual cells as essential features of life [1]. In this respect, the cell adhesion and signaling receptors of the integrin superfamily are thought to play a major role. Integrins are heterodimeric transmembrane glycoproteins, consisting of one α- and one β-subunit. They execute crucial regulatory functions during cell adhesion, migration/invasion, proliferation, survival, and apoptosis. Moreover, integrins are capable of bidirectional signaling across cell membranes, referred to as “outside-in” and “inside-out” signaling, which results in information exchange between the ECM proteins and intracellular molecules [2,3,4,5]. Since integrins are linked to cytoskeletal components within the cell and with ECM proteins within the extracellular space, they function as mechanotransducers exhibiting force-sensing capabilities. These functional features of integrins have gained increasing attention in recent years [6,7].

In 1984, Pierschbacher and Ruoslahti described the Arg-Gly-Asp (RGD) peptide motif as a highly conserved minimal integrin recognition sequence within fibronectin [8]. Subsequently, the RGD motif was identified in many other ECM proteins, including vitronectin [9], von Willebrand factor [10], osteopontin [11], and laminin [12]. Although many integrins recognize ECM proteins via the RGD motif, the specificity of integrins governing this interaction turned out to be more complex. Meanwhile, it became clear that ligand specificity for integrins—besides the RGD motif—also depends on the distinct conformational and spatial presentation of this motif in various ECM proteins as well as on other synergistically functioning adjacent molecular regions [13,14,15]. Based on these findings, synthetic peptides and peptidomimetics, displaying the RGD motif have been developed as potent integrin ligands and antagonists, inhibiting the adhesion of anchorage-dependent cells to ECM proteins and thereby controlling integrin-mediated (tumor) biological functions.

Among the 24 human integrin subtypes known to date, eight integrin dimers, i.e., αvβ1, αvβ3, αvβ5, αvβ6, αvβ8, α5β1, α8β1, and αIIbβ3, recognize the tripeptide RGD motif within ECM proteins. Those members constitute a most important integrin receptor subfamily instrumental in cancer and their metastasis [16]. The exploration of functional differences between distinct members of the integrin subfamily is challenging, because the integrin expression profiles strongly depend on the cell type and the microenvironment within a given temporal and spatial context. To add even more complexity, integrins act in concert with other receptor proteins that are functionally and/or spatially organized within multiprotein networks within so-called focal adhesions (FA) [17,18]. Thus, the elucidation of the specific properties of distinct integrin subtypes governing their in vivo activities requires highly active and selective ligands.

The rapidly growing field of integrins as targets for cancer diagnosis and therapy has stimulated us to provide a thorough update on the impact of RGD-recognizing integrins in cancer, their distinct tumor-cell-specific functions and their crosstalk with other membrane receptors in various cancer entities. For previous reviews of integrins in cancer please see [19,20,21,22,23,24,25,26,27,28].

## 2. Integrin Activation upon Conformational Rearrangements

**Figure 1:**
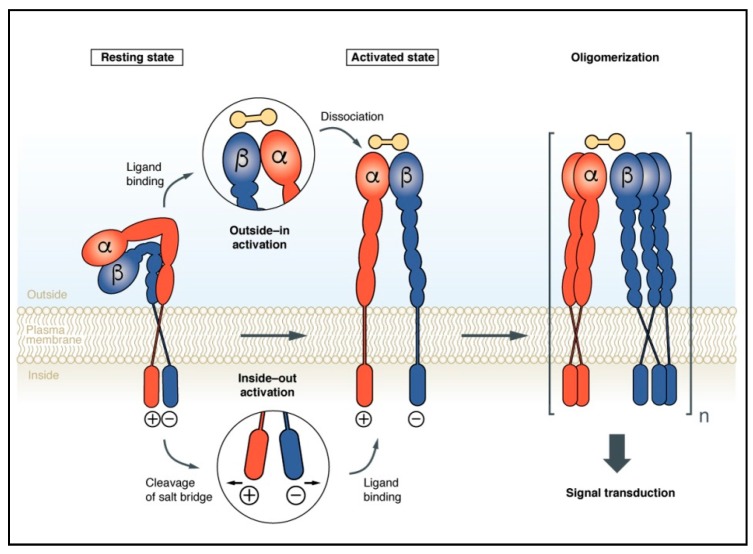
Schematic illustration of integrin activation and the “inside-out” and “outside-in” signaling mechanism. Integrins in the bent resting conformation reveal low affinity binding to their ECM ligands. The inside-out signaling involves disruption of the intracellular salt bridge, which is established between the cytoplasmic subunits. This induces dissociation of the transmembrane helices, followed by the reorganization and generation of a high affinity binding integrin, plus multimerization in focal adhesions. Conformational changes of the resting integrin state are induced by the integrin binding of ECM ligands causing stronger binding at the focal adhesions. The outside-in signaling requires integrin oligomerization [35].

Over the many years following their discovery, integrins have been characterized in great detail. Nevertheless, the exact mechanisms of their molecular activation and bidirectional signaling remained unclear for a long time, mostly because of the missing structural information regarding these non-covalently linked heterodimeric receptors. This changed in 2001, when the structure of the extracellular domain of αvβ3 in the absence or presence of Cilengitide, a small cyclic peptide ligand, was solved [29,30]. These structural data unraveled that the extracellular domains of αvβ3 exist in a resting state in a conformation in which the integrin head groups are bent towards the cell membrane. Ligands may even bind to the kinked integrin in the resting state in which the transmembrane domains of each subunit are closely associated [31,32]. Upon ligand binding, however, structural rearrangements in the hinge region of each integrin subunit occur via the so-called “switchblade” mechanism. Meanwhile, several structural studies have elucidated the details of the conformational alterations during integrin activation [2]. Key elements in the outside-in activation are a shift of the α7-helix in the βI-domain and a dissociation of the transmembrane helices of the α- and the β-subunit [31,33]. Homo-oligomerization of both subunits then activates the intracellular part by the binding of intracellular proteins, such as talin and kindlin, to the cytoplasmic domain of the β3-subunit, which is crucial for outside-in signaling [34,35] (Figure 1). In turn, the binding of talin results in changes within the integrin conformation to an activated and erected state, accompanied by the separation of the integrin transmembrane and cytoplasmic domains (inside-out). The headpieces either remain in the closed and bent state or in the open conformation, which then results in high ligand binding affinity.

**Figure 2:**
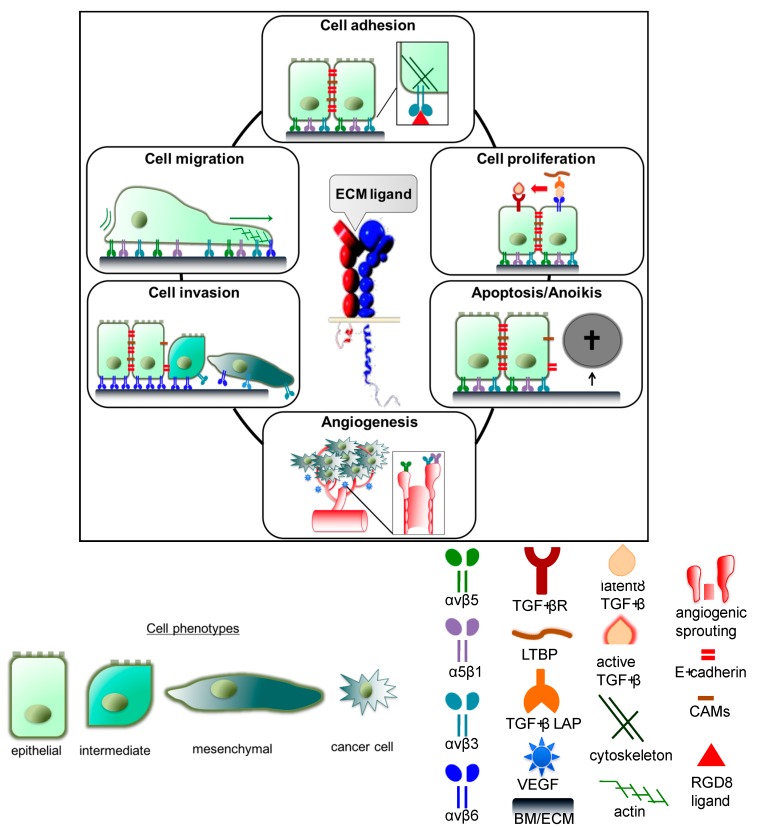
Integrin functions are instrumental in tumor biological contexts. Integrin functions are involved in various tumor biological processes, including cell adhesion, proliferation, inhibition of apoptosis/anoikis, induction of angiogenesis, cell invasion and migration. Adhesion: Cell adhesion is mediated by integrin binding to the respective recognition motif of RGD-ligands within the ECM, e.g., fibronectin, osteopontin and vitronectin. Binding of RGD ligands to integrins enables communication between the ECM and intracellular components, such as the cytoskeleton. Proliferation: Cell proliferation, mediated by integrin subtypes, such as αvβ3, αvβ6, and αvβ8 may also be induced upon the binding of the RGD-containing latency associated peptide (LAP) of the inactive TGF-β to integrins. Force between the TGF-β binding proteins (LTBP) and the TGF-β molecule results in the activation of the latent TGF-β. Subsequent binding of TGF-β to the TGF-β receptor (TGF-β-R) induces epithelial-mesenchymal transition (EMT) and cell proliferation. Apoptosis/anoikis: Further, integrin expression allows cells to bind to ECM molecules within a mesenchymal tissue context, thereby inhibiting apoptosis/anoikis of invasive carcinoma cells. Angiogenesis: Upon tumor growth, the expression of hypoxia induced factors and vascular endothelial growth factor (VEGF) results in the induction of integrin-associated vessel sprouting and angiogenesis. Invasion/migration: The integrin switch from αvβ1 and/or αvβ5 to αvβ6 allows cells to migrate and cross the basement membrane (BM) to invade the surrounding ECM as invasive cancer cells. This process is associated with EMT-like alterations of the cell phenotype. The EMT process involves the downregulation of E-cadherin and cell adhesion molecules (CAMs), promoting cell mobility, cancer progression and metastasis.

The elucidation of the molecular mechanisms of integrin activation reveals that integrins can harbor “on” and “off” states upon conformational rearrangements by switching from a low to a high affinity state. The binding of ECM ligands with high affinity then triggers increased adhesiveness and signal transduction to the cytoplasm in the outside-in direction. To this end, phosphorylation events occur to activate downstream signaling kinases, such as the mitogen-activated protein kinases (MAPK), phosphoinositide kinase (PI3K)/Akt, and extracellular signal regulated kinase (ERK) [31,36,37,38,39], which in turn have an impact on cell proliferation, migration/invasion, and cell survival. Modulations of those integrin-mediated cell biological events are instrumental in malignant cellular transformation and tumor metastasis [40,41,42]. Figure 2 summarizes the various functions of specific integrins in the tumor biological contexts.

## 3. Integrins in Cancer Progression and Metastasis

In a series of various cancer entities, the expression of αvβ3, αvβ5, α5β1, α6β4, α4β1, αvβ6 and αvβ8 has been shown to correlate well with metastasis and poor patient prognosis. Here, αvβ3, αvβ5, and α5β1 are among the most prominent and well-studied integrin subtypes [21]. Table 1 provides an overview of RGD-binding integrins, their expression in various human cancer entities and their prognostic impact.

As such, the enhanced expression of αvβ3 favors tumor growth and invasion and metastasis to the bones, especially when ligated by its major ECM ligands vitronectin and/or osteopontin [43,44]. In addition to αv-integrins, α5β1 expression levels are strongly induced upon hypoxia and promote tumor metastasis in breast cancer. Thus, high α5 expression in clinical biopsies is associated with an increased risk of mortality [45].

Moreover, integrin αvβ6 has been proposed to serve as a marker for tumor cell invasiveness of gastric, pancreatic, cholangiocellular, breast, ovarian, colon, and head and neck cancer. Clinical data have demonstrated that αvβ6 expression levels correlated with poor patient outcome [46,47,48,49,50,51,52,53,54,55]. In accordance, high αvβ6 expression seems to be associated with a more aggressive disease and, consequently, poor outcome and prognosis for patients afflicted with colon cancer [56,57]. The clinical outcome is mainly attributable to the ability of colon cancer cells to metastasize to the liver. In gastric cancer, αvβ6 expression was present in 37% of cases and was predictive of the reduced survival of cancer patients. The analysis of the relationship between αvβ6 expression and the progression of cancer revealed that induced expression of matrix metalloproteases (MMP) and activation of the TGF-β1 pathway are reiteratively activated [53,58,59]. In oral squamous cell carcinoma (OSCC) cells, αvβ6 is highly expressed at the invasive tumor front. In gastroenteropancreatic adenocarcinomas, however, only weak αvβ6 expression was noticed at the cancer cell membranes [51]. In gastroenteropancreatic malignancies, αvβ6 was predominantly expressed in pancreatic ductal adenocarcinomas, followed by gastric carcinomas of the intestinal type, and intestinal adenocarcinomas, without a correlation of αvβ6 expression and its ligands, such as fibronectin and tenascin. Most interestingly, αvβ6 expression was elevated in well to moderately differentiated as compared to poorly differentiated tumors. This observation indicates a possible additional role of αvβ6 during cancer differentiation of gastroenteropancreatic adenocarcinomas [51].

In addition, we wish to stress here, that the integrins αvβ3 and α5β1 work in concert with growth factors and their receptors in order to influence differentiation processes in cancer stem cells in several cancer entities [75,76,77]. Cancer stem cells display increased expression of αvβ3 on their membranes. The ligation by vitronectin results in the loss of nuclear β-catenin and the downregulation of genes that contribute to cancer stem cell maintenance [78]. To this end, strategies to target αvβ3 and downstream signaling molecules, such as K-Ras are currently under investigation with the aim of interfering with cancer stemness [79]. Moreover, αvβ3 represents an indicator for chemosensitivity predicting the response of tumor cells to chemotherapeutics [80,81].

As some malignancies are associated with viral infection, αvβ6 and αvβ8 interestingly plays a crucial role during the entry of the foot-and-mouth-disease virus, the Herpes simplex virus (HSV), and the Epstein-Barr viruses. Virus entry into the cells depends on activated viral glycoproteins. HSV infect cells via species-specific glycoproteins and the conserved apparatus gH/gL and gB. Here, HSV uses αvβ6 or αvβ8 as gH/gL receptors [82]. Furthermore, the fusion of epithelial cells with Epstein-Barr virus proteins can be triggered by the binding of viral glycoproteins gH/gL to αvβ8 [83,84].

In the subsequent sections, we will address the characteristics and tumor biologically relevant functions of specific integrin subtypes and their interactions with distinct signaling networks.

### 3.1. Integrin-Mediated Cell Adhesion, Migration, and Invasion

Epithelial-type, anchorage-dependent tumor cells need to establish adhesive cell-cell and cell-ECM contacts with their microenvironment in order to evade anoikis, a special form of apoptosis attributable to the loss of cell-cell and cell-ECM contacts.

Normal epithelial cells acquire migratory capacities exclusively during embryonic development, tissue renewal and during tissue remodeling, e.g., during wound healing. In contrast, during transformation, tumor cells gain the ability to cross tissue boundaries and to invade into the surrounding ECM and the adjacent vasculature by loosening cell-cell and cell-ECM contacts. This is a prerequisite for the ability of cells to disseminate to distant body compartments and form metastases [85,86]. Hereby, effective cell migration of individual cancer cell types is influenced by the adhesive strength of their cell-cell and cell-ECM contacts [87]. As such, by altering the adhesive strength, the fine-tuned balance between increased and decreased cell motility might be tipped in order to move a cell´s body forward [88]. Hereby, integrins are also involved in cell shape alterations, which, at least in part, depend on integrin clustering and actin filament polymerization at the leading cell front of the migrating tumor cell. This is achieved by integrin cooperation with small guanosine-5′-triphosphate (GTP)-binding proteins, such as Rho and Rac [89].

The acquirement of persistent adhesive and migratory properties involves the epithelial-mesenchymal transition (EMT), which results in the loss of epithelial cell polarity and the formation of an elongated fibroblastoid cell morphology. During EMT, the expression of the cell-cell adhesive protein E-cadherin is decreased concomitant with the elevation of vimentin, desmin, and other mesenchymal markers [90,91].

Breast cancer cells utilize EMT to facilitate their invasion into the vasculature and to establish tumor metastases at distant sites [92]. In the following sections, the most important RGD-recognizing integrin subtypes and their roles in communicating cellular adhesion, migration and invasion, will be considered.

#### 3.1.1. Integrin αvβ3 and α5β1

Integrin αvβ3 was first identified by Ruoslahti and coworkers [93]. Because of its predominant ECM ligand, it was originally named vitronectin receptor. In later studies, however, αvβ3 turned out to be a highly promiscuous integrin that binds to a plethora of different ECM proteins, among them fibronectin, osteopontin, and laminin, via the RGD motif, thereby triggering integrin signaling. The need for αvβ3-provoked signaling in order to induce cell migration has been reported for many different cell types, including smooth muscle cells [94], endothelial cells [95], and various tumor cells [96]. In human ovarian and breast cancer cells, enhancement of αvβ3/vitronectin-mediated cell adhesion was shown to be indispensable for the provision of a sufficient grip of cells to the underlying ECM and the necessary cytoskeletal rearrangements [97,98,99]. Indeed, tumor cells exhibiting high αvβ3 expression levels are capable of protruding broad lamellipodia, associated with decreased RhoA activity [100,101]. With regard to the regulation of cell contractility, cell/ECM stiffness, and cell movements, αvβ3 needs to cooperate with α5β1 in response to applied tension [87,102]. In prostate cancer cells, αvβ3 expression also provoked increased chemokine receptor expression, leading to enhanced migration/invasion during tumor cell metastasis to the bone [103].

Similar observations were made for integrin α5β1. Breast cancer cells with high α5β1 levels have revealed a 3-fold increased cell invasiveness, compared with cells exhibiting low α5β1 expression. However, invasiveness can be reduced by the myosin light chain kinase inhibitor ML-7, only in cells with a high α5β1 expression. This suggests a crucial role for α5β1 in the enhancement of cell migration and invasion by transmission and generation of contractile forces [104]. Moreover, the endocytic recycling of α5β1 also enhances cancer cell invasion, driven by RhoA and filopodial spike-based protrusions [105].

#### 3.1.2. Integrin αvβ6

The expression of αvβ6 is initiated during embryonic development with high levels being exclusively restricted to epithelial cells, the developing lung tissue, and the kidney epithelia [106,107]. In a physiological context, αvβ6 is not constitutively expressed in differentiated epithelial cells, however, it becomes upregulated in the context of tissue remodeling, including wound healing and carcinogenesis [108]. The prevalence of αvβ6 expression has been described in several kinds of malignancies [46].

In addition to RGD-containing ECM ligands, such as fibronectin, tenascin-C, osteopontin, and vitronectin, αvβ6 binds to the RGD motif contained within the latency associated peptide (LAP) of TGF-β to activate the inactive, latent form of the TGF-β molecule. TGF-β signaling has a strong impact on EMT during invasive growth and cancer progression and is a crucial physiological process during embryonic development. In differentiated cells, however, EMT-related cellular alterations have also been observed during fibrotic tissue remodeling, wound healing, invasive cancer growth, and tumor metastasis. One hallmark of EMT is the downregulation of E-cadherin, which provokes loss of epithelial cell-cell adherence, followed by the detachment from the epithelial tissue and increased cell motility [109]. Because αvβ6 functions as a potent activator of TGF-β, a crucial role for αvβ6 expression during the EMT process has been suggested [46]. The activation of TGF-β1 by αvβ6 at the epithelial-mesenchymal interface seems to be a highly conserved EMT mechanism, first established during embryonic development, and then reactivated in the context of carcinogenesis. Cell alterations associated with EMT include a switch from αvβ1 and/or αvβ5 to αvβ6 and render αvβ6 an important EMT-marker for invasive cancer cells [42,110,111]. This switch enables epithelial cells to bind to ECM proteins, to cross tissue boundaries, and to invade mesenchymal tissue. Thereby, cancer cells evade apoptosis (anoikis), which under normal conditions would be provoked because of the loss of cell-cell and cell-ECM contacts [112,113]. Increased expression of αvβ6 in OSCCs triggers EMT, indicated by a decreased E-cadherin expression and the gain of vimentin expression [58]. Among the most common skin cancers, basal cell carcinomas (BCC) are caused by the deregulation of the Sonic hedgehog (Shh) signaling pathway. Although αvβ6 is expressed in low risk BCC, it is markedly upregulated in infiltrative BCC and promotes invasion by modulating the tumor stroma and activating the TGF-β1 pathway [67].

The induction of tumor cell-associated proteolytic activity and the downregulation of E-cadherin, as hallmarks of EMT, promote cancer cell migration, invasion, and tumor metastasis. Hence, the high invasive capacity of αvβ6-positive cancer cells is shown by the observation that αvβ6 expression is correlated with an elevated MMP-9 expression at the invasive cancer front [114]. MMPs are zinc-dependent metallo-endopeptidases involved in the degradation of ECM components [59]. Under physiological conditions, the basement membrane functions as a natural barrier for migrating/invasive cells. Elevated expression of MMP-9 degrades collagen type IV, one of the major components of the basement membrane. In ovarian cancer tissues and cultivated ovarian cancer cells, elevated αvβ6 expression is associated with the secretion of urokinase-type plasminogen activator (uPA), MMP-2, and MMP-9 [49]. Immunoprecipitation studies have revealed that αvβ6 directly interacts with the uPA receptor (uPAR) in ovarian cancer cells to promote cell migration and ERK activation. The interaction of αvβ6 with uPAR is restricted to ovarian cancer cells accompanied with increased TGF-β1 [115]. In OSCCs, ligand binding to αvβ6 leads to the recruitment of the focal adhesion kinase (FAK) and the activation of the Raf-ERK/MAPK pathway, thereby inducing MMPs and promoting cell proliferation and experimental metastasis [116]. Integrin αvβ6 expression in OSCC also activates MMP-3, a crucial factor for ECM degradation and remodeling, the subsequent activation of further MMPs, cell migration, and invasion upon binding to fibronectin [58]. Thus, αvβ6 contributes to the remodeling of the ECM as a prerequisite for cell invasion, migration, and tumor metastasis. These cell-cell and cell-ECM interactions in OSCCs also involve EMT-associated events, such as vimentin expression and E-cadherin downregulation [58]. As in OSCC, invasive endometrial carcinomas have been characterized by elevated αvβ6 at the leading invasive front, concurrently with MMP expression and metastasis formation [70]. Several studies have described the influence of αvβ6 activation/inhibition on the MMP pathway together with its binding of RGD-containing ligands, such as fibronectin, tenascin-C, vitronectin, and the LAP of TGF-β1 or TGF-β3, respectively. Deregulated ECM degradation further promotes invasive growth and malignant progression, as abnormal ECM degradation products bind to integrins, leading to the initiation of integrin signal transduction. For example, the cleavage of fibronectin by MMP-9 results in a 120-kDa fragment that promotes the migration of αvβ6-positive tumor cells. In turn, the increased expression of MMP-9 and MMP-2 leads to a positive feedback loop that might further promote cancer development [117]. At the invasive front of OSCC, MMP-7, -9, -12, and high levels of αvβ6 have been described as prognostic markers [72]. The last 11 amino acids (EKQKVDLSTDC) of the cytoplasmic tail of the β6-subunit seem to be sufficient to activate MMP-9 and promote invasiveness. However, this process depends on specific integrin subunits, because the β3-subunit activates MMP-2 [118]. In OSCC cell lines the migration of αvβ6-positive cells attributable to MMP-9 activation depends on integrin binding to insoluble LAP, whereas soluble LAP downregulates the αvβ6-induced adhesion and migration of OSSC cells [119]. Accordingly, the overexpression of αvβ6 enables cells to migrate onto fibronectin with increased invasiveness because of MMP-9 upregulation [50,120]. The main signaling event, associated with αvβ6-dependent MMP activation involves the ERK/MAPK pathway [121]. Ligand binding of fibronectin to αvβ6 recruits the Fyn tyrosin kinase to the β6-subunit. This activates MMP-3, which, in turn, degrades the ECM. Thus, the interrelation of MMP and integrin expression provides a putative molecular mechanism for αvβ6 enhancement to promote metastasis of OSCC cells. Although the exact molecular mechanisms of the integrin signaling network still remain to be fully elucidated, evidence points to a major role of the ERK/MAPK and TGF-β1 pathway in MMP activation by αvβ6 in various cancer types [116].

A different aspect of integrin function during cancer development is provided by the role of αvβ6 during endocytosis. Its interaction with the hematopoietic lineage cell-specific protein-1 (HS1) associated protein (HAX-1) is responsible for the clathrin-mediated endocytosis of αvβ6, an event that promotes the invasive behavior of OSCCs [122]. A recent study further indicates that the clathrin or non-clathrin-mediated endocytosis of integrins affects cell-signaling pathways that control cancer progression [123]. Consequently, the interaction between HAX-1 and αvβ6 might be the first evidence that integrin-triggered endocytosis plays a crucial role during cancer development and progression.

#### 3.1.3. Integrin αvβ8

A further RGD-recognizing integrin is represented by αvβ8. In accordance with its β6 counterpart, β8-integrin is a 100 kDa protein that exclusively heterodimerizes with the 130-kDa αv-subunit [124]. Integrin αvβ8 is far less studied than other members of the integrin αv-subfamily; however, it is not only structurally, but also functionally related to αvβ6. Nevertheless, its role in adhesion, invasion and migration remains to be clarified.

Its biological functions are mainly associated with its action as a potent activator of TGF-β1 [125]. Loss-of-function experiments have revealed that mice lacking αvβ6 and αvβ8 activity reproduce the abnormalities observed in TGF-β1 and TGF-β3 null mice [126]. Site-directed mutagenesis of latent TGF-β1 has demonstrated that the high affinity binding of αvβ8 to latent TGF-β1 is defined by Leu-218 following the RGD motif contained within LAP [127]. In contrast to αvβ6, αvβ8 has a shorter cytoplasmic tail and does not bind to actin. Therefore, the αvβ8-dependent activation of latent TGF-β1 from the large latent complex (LLC) have been suggested not to work by force, but to require proteolytic cleavage by co-expressed MMP-14 (or MT1-MMP) [128]. The control of the subsequent intracellular transduction of αvβ6 signaling is gradually being elucidated. So far, the Band 4.1B and the Rho GDP Dissociation Inhibitor 1 (RhoGDI1) have been identified as β8-binding proteins [129] and the cytoskeletal adapter protein spinophilin has been described to regulate tumor cell invasion in glioblastomas [130]. Interestingly, during wound healing, TGF-β is activated via αvβ8 and αvβ6. In contrast to αvβ6, the inhibition of αvβ8 enhances the degree of cell motility [131].

Moreover, αvβ8 plays a dominant role in promoting the migration of astrocytes onto vitronectin [132]. Astrocytic αvβ8 expression has been proposed to act as a central regulator of brain vessel homeostasis because of the regulation of TGF-β activation and downstream genes that promote vessel differentiation and stabilization, such as the plasminogen activator inhibitor-1 (PAI-1) and thrombospondin-1 [133].

### 3.2. Impact of Integrins on Cellular Proliferation

In normal cells, distinct mechanisms regulate and terminate integrin-triggered signaling pathways to keep cell biological events under a balanced control and to maintain cells anchored onto the ECM in a quiescent state. Under normal conditions, integrins are essential players for the maintenance of tissue integrity. In contrast, tumor cells are characterized by uncontrolled behavior and acquire self-sufficiency regarding the expression and action of growth factors and their receptors, cytokines, and the activation of oncogenes, leading to the loss of control over cell proliferative activity in their pericellular microenvironenment. Hanahan and Weinberg have defined the hallmarks of cancer as (i) continuous proliferation, (ii) self-sufficiency, (iii) invasion and metastasis, (iv) limitless replicative potential, (v) promotion of angiogenesis, and (vi) evasion of apoptosis [134].

#### 3.2.1. Integrin αvβ3

The growth-promoting effects of αvβ3 on breast and prostate cancer cells and on malignant melanoma have been well characterized [20]. In ovarian cancer, αvβ3 is instrumental in increasing cell proliferation by means of signaling that involves integrin-linked kinase [99]. Consequently, the blocking of αv results in drastic cell cycle arrest [135].

During their functions in cell proliferation, integrins crosstalk with growth factors and their respective receptors [136]. In breast and pancreatic cancer as well as glioma cells, αvβ3 physically and functionally cooperates with, for example, the epidermal growth factor receptor (EGF-R), Erb-B2, and the platelet-derived growth factor (PDGF-R) whose activation drives cell proliferation [137,138,139]. In ovarian cancer cells, αvβ3 expression correlates with enhanced expression and activity of the EGF-R [140]. In this way, even in the absence of respective ligands, growth factor receptor and integrins synergistically induce cellular signaling cascades. In cancer cells, constitutively activated integrin signaling not only leads to dysregulated tumor cell growth, but also avoids apoptosis. The effects of induced cell proliferation by αvβ3 is even further enhanced by the action of anti-apoptotic proteins Bcl-2 and FLIP [90].

TGF-β signaling is well known for its anti-proliferative effects. However, its role in tumor biology is heterogeneous and depends on the oncological context. TGF-β function can switch from inhibition of cell proliferation as a tumor suppressor to activation of a pro-oncogenic EMT-program. The effect of TGF-β signaling is influenced by the abundance of active TGF-β molecules. Here, RGD-binding integrins play a crucial role by binding to LAP and activating the latent form of the TGF-β molecule. The consequences will be explained and exemplified in the following based on the prevailing data for αvβ6 [125].

#### 3.2.2. Integrin αvβ6

The binding of LAP to αvβ6 releases and activates the inactive pro/LAP-TGF-β [141,142]. Hence, αvβ6-positive cancer cells become self-sufficient in growth signals. In a study of cervical cancer patients, those with positive αvβ6 expression had different expression levels of p53, PCNA, Ki-67, and TIPE2 as proliferation markers. Thus, αvβ6 was described to cause active proliferation but to inhibit apoptosis, at least in cervical cancers [143].

#### 3.2.3. Integrin αvβ8

In contrast to the effects of αvβ6 on pro/LAP-TGF-β, the activation of TGF-β by αvβ8 has interestingly been described to inhibit the proliferation of the airway epithelium in intact bronchial tissue [144]. In this manner, the β8-subunit seems to regulate the growth of epithelial cells and has been suggested to inhibit tumor growth in nude mice [145]. Interestingly, integrins seem to play heterogenous roles in regulating cell proliferation: growth-promoting integrin signals may be counterbalanced via inhibitory integrin signaling pathways, which can be activated by cytoplasmatic integrins domains as the results of either alternative splicing or evolutionary divergence [146].

Integrin αvβ8 has been suggested to regulate proliferation negatively for three main reasons. First, the cytoplasmic part of the β8-subunit differs in sequence, compared with other strongly related cytoplasmic domains of αv-associated integrin subunits, such as β1, β3, β5, and β6. Second, the β8 cytoplasmic domain cannot support stable, high-affinity adhesion to vitronectin, and third, β8 expression is restricted and mainly upregulated in non-proliferating cell types [145]. However, the overexpression of αvβ8 facilitates the proliferation and invasion of OSCC cells via the MEK/ERK signaling pathway upon the binding of αvβ8 to collagen type-1 [147].

In the context of chronic obstructive pulmonary disease (COPD), TGF-β activation via αvβ8 is known to enhance interleukin (IL)-1β-dependent fibroblast expression of the ccr6 chemokine ligand ccl20, suggesting that αvβ8, ccl20, and ccr6 interaction leads to the accumulation of dendritic cells (DC) around airways [148]. The convergence of the TGF-β and the IL-1β signaling pathways on the ccl20 promoter has been defined as a mechanism by which the αvβ8-mediated activation of TGF-β regulates IL-1β-dependent ccl20 expression in COPD patients [149]. The αvβ8-mediated activation of TGF-β regulates the chemokine secretion of lung fibroblasts, which directs DC and regulates fibrotic and immune responses in the lung [150]. Interestingly, T regulatory cells (tTregs) lacking αvβ8 are unable to suppress pathogenic T-cell responses during active inflammation; however, the deletion of αvβ8 does not result in a spontaneous inflammatory phenotype [151]. For thymus-derived tTregs, αvβ8 is referred to as a marker protein that mediates the processing of latent TGF-β1 from the latent TGF-β1/glycoprotein-A repetitions predominant protein (GARP) complex on the surface of tTregs [152].

### 3.3. Integrin Effects on Cell Survival and Apoptosis

The integrin-mediated anchorage of epithelial-type cells to the ECM is instrumental for maintenance of cell viability via the activation of FAK-, the PI3K/Akt, and the FAK-MAPK-pathways. In contrast, unligated integrins may eventually lead to cellular apoptosis (integrin-mediated death) [153,154]. Tumor cells derived from epithelia, even losing their ECM contacts, have the ability to overcome controlled cell death (anoikis), e.g., by increasing the expression of receptor tyrosine kinases or small guanosine-5′-triphosphate (GTP)ases in concert with downregulated caspase 8 expression [155,156]. Moreover, tumor cells may circumvent anoikis by modulating the expression pattern and cell surface density of their integrin subtypes and by internalizing activated integrins with sustained signaling in endosomes [111,157,158]. Cancer cells also manage to escape from anoikis by integrin crosstalk with growth factor receptors, resulting in effective survival signaling [159,160]. Interestingly, αvβ3-expressing tumor cells, which are detached from the ECM, nevertheless exhibit enhanced tumor growth in vitro in an anchorage-independent fashion. The growth-promoting effect depends on the recruitment of c-Src to the β3-integrin cytoplasmic tail. This, in turn, activates c-Src, leads to the phosphorylation of the Crk-associated substrate (CAS), and promotes tumor cell survival in a mechanism independent of FAK activation. These findings have unraveled an interesting role of αvβ3 in anchorage-independent tumor cell growth and aggressiveness [21]. Loss of normal integrin action may also arise from abnormal integrin internalization. To this end, β3-integrin has been reported to associate with caveolin-1, indicative of raft/caveolar endocytosis. Following cell detachment, lipid rafts are rapidly internalized. This, in turn, inhibits key integrin signaling molecules such as Erk, PI3K, and Rac. In cancer cells, however, this process appears to be hindered by β3-integrin whereby integrin signaling is maintained [21]. In glioblastoma cells, which overexpress αvβ3 at the invasive tumor front, fibronectin expression is elevated accompanied by increased cell motility and resistance to apoptosis. Subsequently, it has turned out that apoptosis in glioblastoma cells is not regulated by integrins alone but by their interconnections to the p53 pathways [161,162]. It is observed that integrin α5β1 is more important for inducing apoptosis in glioblastomas than αvβ3 [161,162]. Loss of anchorage-dependence and consequently, resistance to anoikis, are extremely important features during malignant transformation of tumor cells, enhancing tumor development and metastasis [155,163].

### 3.4. Integrins αvβ3, αvβ5, and α5β1 in Tumor Angiogenesis

Tumor cells, like normal cells, strongly depend on the continuous supply of nutrients and oxygen in order to maintain their vital features. Further outgrowth of tumors above a tumor size of approximately 2 mm in diameter is largely restrained by the absence of functional blood vessels in the tumor vicinity. Therefore, tumor cells are forced to induce the formation of new vessels, an event involving the three endothelial integrins, αvβ3, αvβ5, and α5β1 [164,165]. This is achieved by the secretion of pro-angiogenic molecules, such as the vascular endothelial growth factor (VEGF) and the fibroblast growth factor-2 (FGF2), which upon engagement of their respective receptors leads to the so-called “angiogenic switch” [166]. VEGF-mediated angiogenesis occurs via αvβ3, whereas FGF-2-mediated angiogenesis is mainly triggered by αvβ3 and α5β1 [167]. Signaling by these receptor/ligand systems contributes to the formation of new vessels, derived from the surrounding pre-existing vasculature. Hereby, per se quiescent endothelial cells are activated to proliferate and migrate towards the tumor site. This motile activity depends on the remodeling of the ECM, predominantly provoked via proteolytic ECM breakdown by MMPs and uPA. Integrin αvβ3 co-localizes and assists during the activation of MMPs on the surface of angiogenic blood vessels. This provokes the remodeling of collagen and the basement membrane. The consecutive ECM breakdown then facilitates endothelial cell migration [168,169]. In accordance, pancreatic tumor cells have been shown to exhibit increased αvβ3 levels that correlated with enhanced MMP-2 activation and lymph node metastasis [65].

One predominant trigger for tumor angiogenesis becomes relevant as soon as tumor growth exceeds blood supply and causes a hypoxic gradient. Hypoxia induces the translocation of the hypoxia-inducible factors-1 α (HIF-1α), HIF-2α, and HIF-3α into the nucleus. Here, they recognize hypoxia response elements (HREs) and induce gene transcription upon binding to the respective promoter regions of their many target genes, including αv, β3, β1, and β6, and the integrin-linked kinase (ILK) and VEGF [170,171]. In turn, these integrins enhance a migratory and invasive tumor cell phenotype that establishes a positive feedback loop and furthers EMT and tumor metastasis [172,173,174,175,176,177,178,179].

In animal models, the blockade of αvβ3 and αvβ5 by specific peptide antagonists, such as Cilengitide, or by blocking antibodies, such as Vitaxin, results in the drastic reduction of angiogenesis. This blockade also inhibits downstream signaling via the FAK/Src/Akt-pathway, leading to endothelial and tumor cell apoptosis [180]. Cilengitide is effective in combination with chemotherapy [181]. However, studies in αv-, as well as β3/β5-knock-out mice indicate that αvβ3 is important but not indispensable for tumor angiogenesis. In addition, β5 does not represent an absolute requirement for this process, because extensive angiogenesis and tumor growth were still observed [182,183,184]. Thus, α5β1 has been proposed also to take over as yet not fully resolved functions during angiogenesis, especially since Hynes and co-workers have shown that αv and α5 cooperate and are capable of functionally backing each other up [185]. Interestingly, low dose administration of the αvβ3 ligand activated this integrin [182,186,187] and opens a new way in stabilization of the vascular system in the treatment with cytotoxic drugs [188].

As regards αvβ8 expression, it activates a TGF-β gradient in the brain, a gradient that is angio-suppressive and inhibits endothelial cell sprouting. Consistently, the loss of αvβ8 in the brain or downstream TGF-β1 signaling via ALK5-Smad3 in endothelial cells increases vascular sprouting, branching, and proliferation, resulting in vascular dysplasia and hemorrhage [189]. However, in a developmental context, αvβ8, βPix, and GIT1 have been suggested to regulate vascular stability, cerebral angiogenesis, and endothelial cell proliferation in the developing embryo [190]. In the developing retina and on astrocytes αvβ8 expression is essential for neovascularization and regulates blood vessel sprouting [191]. Astrocytoma cells selectively suppress αvβ8 expression to manipulate their antigenic balance in order to exploit the signaling pathways of developmental brain angiogenesis in adult brain tumors [192]. The reduced expression of the β8-chain had been considered to be instrumental for the pathogenesis of sporadic brain arteriovenous malformations [193].

### 3.5. TGF-β1 and Its Integration with Integrin Signaling

One major inducer of EMT is TGF-β, which is produced at high levels in breast, prostate, and other cancer cells [194]. The TGF-β family comprises three isoforms: TGF-β1, TGF-β2, and TGF-β3. Each member is processed as a homodimeric pro-TGF-β. This cytokine is deposited in the ECM in an inactive form linked to LAP and one of four latent TGF-β-binding proteins (LTBP) [195]. Upon the binding of αvβ3, αvβ6, or αvβ8 to the RGD motif contained within LAP, a conformational switch is provoked, leading to the exposure of TGF-β1 to the neighboring cells where it ligates its respective receptors. This provokes the downregulation of E-cadherin and increases the expression of mesenchymal proteins, such as N-cadherin [196].

Pro-TGF-β1 monomers contain an aminoterminal pro-domain of 249 amino acid residues, separated by a pro-protein convertase cleavage site from the 112-amino acid residue carboxy-terminus of the TGF-β1 domain. It is cleaved intracellulary by furin-like enzymes and remains non-covalently linked to LAP in order to form the small inactive latent complex (SLC) [197]. The binding of LAP to the mature TGF-β molecule prevents signaling through the TGF-β-receptors [198]. In order to enable receptor binding, TGF-β1 has to be activated and released from this complex. However, TGF-β1 is mostly secreted as a large latent complex (LLC) formed by the dimerization and covalent disulfide linkage of the LAP-TGF-β to LTBPs or GARP [199]. Each pro-domain forms a ring around TGF-β to keep it inactive/latent [200]. LTBPs belong to the LTBP/fibrillin protein family, comprising fibrillin-1-3 and LTBP1-4 [201]. Except for LTBP3, the LTBP-isoforms LTBP1, LTBP2, and LTBP4 can bind to all TGF-β isoforms. LTBPs have a binding capacity towards fibronectin and vitronectin, thus in the LLC, TGF-β is bound to ECM proteins. This complex building provides in the ECM a pool of latent TGF-β that is activated in a context-dependent manner. In contrast to other mechanisms, such as proteolysis, this way of TGF-β1 activation requires cell traction forces and the association of the LLC with the ECM [202]. Thereby, the binding of αvβ6 to LAP directly influences the migration of cancer cells. A recent crystal structure analysis of the αvβ6 head group bound to pro/LAP-TGF-β1 has provided insights into the interaction between integrins and their macromolecular ligands. Furthermore, it elucidated the way in which integrin binding to ECM molecules transmits activating forces with biological consequences, such as TGF-β1 activation [7]. Interestingly, the resulting effects on the integrin domains by small molecules and RGD-containing peptide binding depend on the force vector and the physiological orientation of the integrin dimer and their ligands.

The activation mechanism of TGF-β1 by αvβ6 has been thoroughly studied [119,141,203]. Upon binding of αvβ6, αvβ3, or αvβ8 to the RGDLXX(I/L) motif within LAP of pro-TGF-β1, these integrins associate with the actin cytoskeleton. This triggers conformational changes in the LLC complex (LAP-TGF-β-LTBP1), releasing TGF-β1 from the LLC and enabling TGF-β1 binding to its receptor [198,199]. Interestingly, TGF-β1 activation by integrins is LTBP-specific, because αvβ6 can only activate TGF-β molecules that are bound to LTBP1 but not to LTBP3. The various isoforms of the LTBPs exhibit significant differences in the sequence of the hinge region, suggesting a functional role in the specific activation of TGF-β1 by the various integrin subtypes [203]. Although LAP-TGF-β1 also binds to other integrins, such as αvβ1 and αvβ3, only αvβ6 can release and activate TGF-β1 from the LLC [204]. However, the mere binding of integrin to LAP is not sufficient to release and activate TGF-β1 in vivo [200]. This is explainable by the different cytoskeleton-binding properties that influence the binding of the cytoplasmic integrin tail to actin and exert traction forces. Recent data have revealed that traction forces by αvβ6 on pro/LAP-TGF-β1 is required for TGF-β1 activation: truncation of the cytoplasmic domain of the actin-binding β6-subunit or deletion of the binding site of the pro-domain of LAP-TGF-β1 to the ECM inhibits the exertion of tensile force across the pro-domain and thus TGF-β1 activation [205].

Upon TGF-β1 activation, the successive signaling events in turn lead to the additional expression of αvβ3 and αvβ6 and of ECM protein ligands, followed by the activation of the PI3K-, Akt, and NF-κB pathways. Moreover, growth factor receptors, such as the EGF-R, are upregulated upon TGF-β signaling; this is of note here, since the EGF-R physically and functionally crosstalks with integrins in a synergistic fashion [127,204,206,207,208,209,210,211,212].

## 4. Challenges for the Design of Novel Integrin Ligands and Their Translation into Clinical Applications

**Figure 3:**
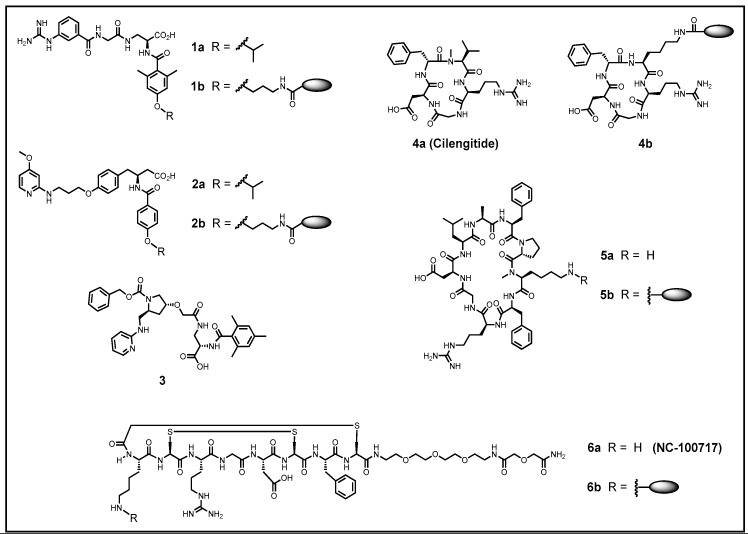
Development and testing of selective integrin ligands. Depicted are some integrin-specific ligands according to their selectivity profile (IC_50_-values) for the subtypes αvβ3, αvβ5, αvβ6, αvβ8, α5β1, and αIIbβ3 [214]. The binding affinities of the ligands have been determined in a cell-free, enzyme-linked immunosorbent assay (ELISA)-like assay for comparability. None of the selected compounds has significant affinity for the platelet integrin αIIbβ3. Specificity or subtype with the lowest IC_50_-values are highlighted. The preferential site for modification with diagnostic or therapeutic agents is indicated by an ellipsoid tag.

The great discovery that the small tripeptide sequence RGD is sufficient to inhibit the interaction of ECM proteins with integrins [8] has stimulated the search for small peptidic or peptidomimetic molecules to selectively address various integrin subtypes and thus their pathophysiological features with high affinity [27,28,213,214]. This culminated in the development of the peptidic compound Cilengitide (4a) (Figure 3) [215,216], which was tested in phase III clinical studies for the treatment of glioblastomas [217,218]. The failure of these studies was a backlash for the development of integrin ligands [219]. However, recent investigations into the mechanism and details of the activities of the various integrin subtypes and their role in apoptosis have shed light on the reasons for this failure and have opened new exciting applications for this molecule [182]. The binding of small ligands can occur to the resting state of an integrin receptor leading to its activation. However, a stronger binding to the focal adhesion complex requires higher concentrations of specific ligands [186].

## 5. Improving the Activity and Selectivity of Integrin Ligands 

Although the above-mentioned integrins all recognize RGD, they differ in their binding activity towards different ECM proteins. Integrins of the αv-family are well known for their strong binding to vitronectin [225]. Very early, the activity and selectivity of peptide ligands to the different integrin subtypes was observed to be controllable by cyclization [226,227], RGD flanking residues [228], the chirality of the amino acids, and the N-methylation of peptide bonds [215,226,227,228,229]. The substitution of arginine by lysine, which removes ligand binding to all αv-integrins, retains, however, the activity for the platelet receptor αIIbβ3 [27,230]. This knowledge and the structural data concerning integrin head groups with and without a ligand have enabled the development of many derivatives with improved integrin binding properties, including peptide mimetics and finally have also led to non-peptidic ligands. X-ray structures of αvβ3 [29,30], αIIbβ1/αIIbβ3 [231], α5β1 [232], and αvβ6 [7] have facilitated the rational design and optimization of ligands. For this aspect of integrin ligand design, please refer to previously published literature [27,213,214]. These selective ligands for distinct integrin subtypes bear the potential of serving as promising new drug candidates for personalized medicine in various cancer entities. Figure 3 depicts a few selected integrin ligands resulting from an extensive effort to develop RGD-peptides or low-molecular RGD-mimicking peptidomimetics, featuring high stability for diagnostic and therapeutic in vivo applications. To this end, the ligands have to be functionalized which is frequently accompanied by a decline in integrin-binding affinity. Hence, after functionalization, the binding affinity of a newly developed integrin ligands has to be carefully controlled and may be minimized by a well-considered selection of the labeling site and an appropriate spacer moiety being placed in between [214,215,220,221,222,223,224,233].

Moreover, during ligand development, we must avoid making the specificity of integrin ligands to be used for therapeutic purposes towards the platelet integrin αIIbvβ3 too narrow, since their systemic administration might lead to major hemorrhagic disorders [26,27,28].

## 6. In Vivo Targeting of Integrins for Cancer Imaging and Therapy

Because of their different functions in cancer biology and the availability of small molecule ligands, RGD-binding integrins have been identified as attractive in vivo targets for the molecular imaging of tumors [107]. The respective contrast agents are usually based on the peptidic or non-peptidic integrin ligands, which are conjugated to suitable labels, e.g., fluorophores, radionuclides, or MRI contrast agents, such as gadolinium complexes. Moreover, integrin ligands have been incorporated into larger structures, e.g., by grafting them onto the surface of nanomaterials, or by their inclusion into liposomes [234,235,236].

### 6.1. Integrin αvβ3 and α5β1

A glance at the currently available literature reveals that the overwhelming majority of pertinent studies is focused on αvβ3. Targeted probes comprising αvβ3-selective cyclic pentapeptides of the RGDxK (x = f, y) type are popular, most likely because the c(RGDxK) motif can be relatively easily synthesized. Thus, they quickly entered the portfolios of producers of fine chemicals as an off-the-shelf building block. Mice models for αvβ3-expressing tumors, such as subcutaneous U87MG glioma or M21 melanoma xenografts, are routinely generated. The imaging of αvβ3 in rodents by using modified RGD peptides has evolved into a standard benchmark scheme for in vivo feasibility studies and the validation of novel bioconjugation and labeling approaches. Hence, a vast number of αvβ3-targeting probes has been described, whereas only a small fraction has actually been pursued beyond the proof-of-principle stage. Notwithstanding this, radiolabeled probes for mapping of αvβ3 expression are widely considered to possess significant clinical potential for tumor imaging. The encouraging results of clinical applications of the first c(RGDfK)-based positron emission tomography (PET) radiotracer ^18^F-galacto-RGD in cancer patients have triggered the development of numerous αvβ3-targeted radiopharmaceuticals for imaging of tumor angiogenesis [237,238,239]. A recent review by Chen et al. [240] summarizes human PET images and additional data for eight different compounds [241,242,243,244,245,246,247,248], some of which are currently in clinical trials, highlighting their capability to visualize solid tumors. However, as has been repeatedly pointed out, the initially envisaged purpose of imaging αvβ3, i.e., the in vivo quantification of the tumor angiogenesis and/or the patient stratification for antiangiogenic therapies, is somewhat thwarted by the finding that αvβ3 expression does not necessarily correspond to angiogenic activity in (tumor) tissues [165,240,249]. Many tumor cell types display membraneous or cytoplasmic αvβ3 expression, but also substantial physiological expression is observed in many organs. As a result, the clinical value of the in vivo mapping of αvβ3 remains to be defined [249].

In contrast to αvβ3, which is not strictly required for tumor angiogenesis [183,184,185], α5β1 suggests itself as a more attractive target, because of the evidence for a stricter link between angiogenesis and β1 integrin expression [250]. Although α5β1 is only weakly expressed in quiescent murine and human endothelial cells [251], it is upregulated on endothelial cells during vessel sprouting during the tumor angiogenic process [167]. Despite this promising perspective, only few imaging probes for α5β1 have been described so far. The first in vivo imaging of α5β1 expression was performed by using ^68^Ga-labeled derivatives of a α5β1-selective peptidomimetic [252,253], followed by radiolabeled derivatives of a linear peptide derived by phage display [254] and of a cyclic peptide comprising the HisoDGR structural motif [255]. The best sensitivity and contrast for α5β1 during PET imaging was hitherto achieved by using ^68^Ga-Aquibeprin [256,257], a trimer of the aforementioned peptidomimetic [252] with an α5β1 affinity (IC50) of 80 pM. However, to the best of our knowledge, no α5β1 imaging in humans has been reported, as yet.

Although in the context of molecular imaging, the ubiquity of c(RGDxK)-based probes has led to the common perception of an equivalence of integrin expression and angiogenesis, this distorted notion might soon be adjusted by the recent advent of imaging agents, targeting αvβ6 [258].

### 6.2. Integrin αvβ6

Several characteristics qualify αvβ6 as a potential diagnostic and therapeutic target. First, it is not constitutively expressed under physiological conditions in differentiated adult epithelia but within a specific pathological context upon cellular alterations going along with EMT, i.e., wound healing, carcinogenesis, and tumor metastasis. Second, the use of targeting diagnostic and therapeutic tools should allow a clear demarcation between healthy and diseased tissue. As an EMT marker, αvβ6 is specifically expressed at the epithelial-mesenchymal boundary and thus enables the specific targeting of invasive and migrating cells. Consequently, because of its high affinity and its *de novo* expression in cancer tissue, its involvement in cellular invasion and metastasis, and its expression in fibrotic tissue, αvβ6 represents a promising cancer cell target [47,58,259]. To this end, increasing efforts are currently being undertaken to synthesize—besides ligands for targeting other integrins—αvβ6-specific peptidic and peptidomimetic integrin ligands/antagonists [260,261,262,263,264]. For the diagnostic imaging of human cancer cells, the peptide A20FMDV2, derived from the food-and-mouth disease virus sequence [262], the peptide H2009.1 [263], and the cyclic peptide S02 [264] are currently being explored for their use as radiolabeled integrin ligands. The idea of targeting RGD-binding integrins for tumor imaging has been followed extensively, however, mainly for αvβ3 [265]. Nevertheless, αvβ6 has been imaged in vivo by single-photon emission computed tomography (SPECT) [258,259,260,261,262,263,264,265,266,267,268] and PET [262,269,270,271,272,273].

**Figure 4:**
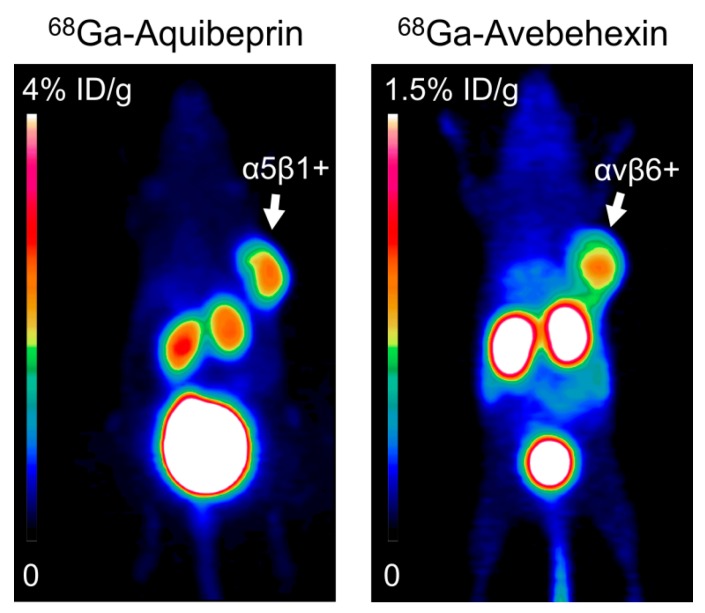
Imaging of various integrin subtypes.

To date, a series of various αvβ6-targeting tracers have been developed for diagnostic purposes, including linear 10- to 20-mer peptides and “stapled” cystine peptides [266,267,268]. Our group have developed and tested enzymatically stable cyclic peptides as novel αvβ6 ligands, revealing so far the lowest molecular weight of all αvβ6-binding molecules with sub-nanomolar binding affinity [223]. Recent work has demonstrated that these novel ligands can serve as the basis for the synthesis of promising new PET-tracers [273]. ^68^Ga-Avebehexin [271], a ^68^Ga-labeled derivative of the metabolically stable cyclic nonapeptide cyclo-(FRGDLAFp(NMe)K) [223], has shown the most advantageous tumor-to-background contrast because of excellent renal clearance (Figure 4). Especially for cancer entities, which exhibit high αvβ6 expression in more than 95% of cases, like in OSCC, its imaging encourages a novel and most promising tool for cancer diagnosis [47,262]. 

Until now, only a single study on αvβ6 targeting carried out in living human subjects has been reported. Using the ^68^Ga- and ^177^Lu-labeled compound SFITGv6, which comprises the binding sequence FRGDLMQL, Altmann et al. performed PET/CT scans of head and neck squamous cell carcinoma (HNSCC) and non-small-cell lung carcinoma (NSCLC) patients and found that the tracer accumulated specifically in tumors, but not in inflammatory lesions [274]. Finally, αvβ6-targeted imaging and therapy has been repeatedly pointed out to hold greatest promise for pancreatic carcinoma (PDAC) [51,73,267,269,275], evidence in humans is still lacking.

As examples, Figure 4 shows µPET images (maximal intensity projections) of SCID mice bearing subcutaneous tumor xenografts on the right shoulders (positions indicated by arrows). Left: M21 human melanoma with high α5β1 expression, imaged using ^68^Ga-Aquibeprin [256,257]. Right: H2009 human lung adenocarcinoma with high αvβ6 expression, imaged using ^68^Ga-Avebehexin [73,275].

## 7. Integrins in Cancer Therapy

Binding molecules with high affinity and selectivity against specific integrin subtypes can serve as key pharmacological tools for studying the biological functions of integrins. We suggest that a detailed analysis of the correlation of integrin subtype expression with cancer progression and the understanding of the underlying molecular mechanisms will help to generate molecular diagnostic and therapeutic tools to improve cancer patient care.

This knowledge will provide the basis for the clinical translation to diagnostic and therapeutic applications. Therefore, increasing efforts are currently being made to synthesize also meanwhile αvβ6-specific non-peptidic and peptidic integrin ligands with antagonizing/inhibiting effects [260,261,262,263,264].

The interruption of integrin-specific functions and signaling by specific integrin ligands has been considered as a promising potential therapeutic approach. This aspect will be exemplified here for αvβ6: the US patent 7150871 describes the successful reduction of metastasis in αvβ6 overexpressing lung cancer cells upon the specific binding of the αvβ6-specific function blocking monoclonal antibody 10D5 [47]. Moreover, in colon cancer cells, this antibody provokes increased cellular apoptosis, accompanied by enhanced ERK phosphorylation [276]. The binding of 10D5 to β6 has been suggested to disrupt binding to ERK and thus reduce metastasis. The major capsid protein VP1 of the O1 strain of the foot-and-mouth-disease virus is well known as a high affinity ligand for αvβ6. A 17-mer peptide derived from the viral coat protein-1 VP1 has been used to create the humanized single chain antibody scFv B63 that can be used to interfere with αvβ6-mediated cancer cell invasion [277]. As described above, small molecule peptidic and peptidomimetic integrin ligands/antagonists are also being explored for their efficacy in inhibiting specific integrin functions, tumor progression, and metastasis. Moreover, the inhibition of the binding of LAP-TGF-β1 to αvβ6 may represent a specific and context-dependent therapeutic approach for αvβ6-positive neoplasia.

## 8. Conclusions

The context-specific expression and selective functions of RGD-recognizing integrins open the possibility for targeting distinct physiological and pathological processes, including cell proliferation, differentiation, apoptosis, adhesion, and migration. All these processes are involved in cell invasion and angiogenesis. Selective ligands for the various integrin subtypes are the key for targeted diagnostic approaches, such as molecular imaging and drug-based therapeutic approaches. Potential clinical applications range from oncologic, to fibrotic and to inflammatory diseases management. Future studies in translationally orientated pre-clinical models and clinical trials will provide insights as to which application might be of clinical relevance. Current clinical trials are addressing the detection of integrin αvβ6 in pancreatic cancer to evaluate the feasibility of [^18^]FP-R01-MG-F2 PET/CT imaging in patients afflicted with pancreatic cancer (NCT02683824) [278]. A further study will provide data considering the side effects of ^18^F-αvβ6-binding-peptide and its feasibility for imaging in patients with primary tumors or cancer that has spread to the breast, colon, lung, or pancreas, in order to improve the detection of the cancer location within the body (NCT03164486) [279].

## Figures and Tables

**Table:** 

	αvβ3 [nM]	αvβ5 [nM]	αvβ6 [nM]	αvβ8 [nM]	α5β1 [nM]	αIIbβ3 [nM]	Ref.
**1a**	>10,000	>10,000	433 ± 101	37 ± 3	2.3 ± 0.02	>10,000	[220]
**2a**	0.65 ± 0.05	199 ± 21	>10,000	>10,000	108 ± 27.5	>10,000	[221]
**3**	>10,000	>10,000	23 ± 3.4	8.2 ± 0.52	2.5 ± 0.4	>10,000	[222]
**4a**	0.61 ± 0.06	8.4 ± 2.1	2050 ± 640	2350 ± 438	14.9 ± 3.1	5400 ± 814	[216]
**5a**	1200 ± 240	>10,000	0.28 ± 0.019	24 ± 3.1	73 ± 6	>10,000	[223]
**6a**	1.1 ± 0.1	16.7 ± 2.1	>10,000	>10,000	820 ± 156	>10,000	[224]

**Table 1:** Overview table of RGD-binding integrins expressed in various human cancers at the tissue level with respect to the expressing cell type and, if available, the prognostic impact.

Integrin	Cancer Type	Cell Type	Main Expression Feature	Reference
αvβ3	gastric cancer	tumor, endothelial and stromal cells	low to moderate expression frequency in tumor cells, high frequency in stroma and endothelia, correlates with phenotype, endothelial expression correlates with survival	[60]
	glioma	endothelial and tumor cells	expression correlates with grade	[61]
	lung cancer brain metastases	endothelial and tumor cells	high expression frequency in endothelial, low frequency in tumor cells	[62]
	non small cell lung cancer	endothelial and tumor cells	high expression frequency in endothelia, low frequency in tumor cells, no correlation with survival	[63]
	oral squamous cell carcinoma	endothelial cells	higher expression in intratumoral endothelia compared with control tissue	[64]
	pancreatic cancer	tumor cells	moderate expression frequency, involved in lymph node metastasis	[65]
	prostate cancer	endothelial cells	high expression frequency peritumoral	[66]
αvβ5	gastric cancer	tumor, endothelial and stromal cells	moderate (to high) frequency in tumor cells, high frequency in stroma and endothelial cells, independent prognostic factor in intestinal-type	[60]
	lung cancer (with brain metastases)	vessel endothelia and tumor cells	high expression frequency in endothelia, low frequency in tumor cells	[62]
	non small cell lung cancer	tumor cells and stroma	high frequency in tumor and stroma cells, no correlation with survival	[63]
	oral squamous cell carcinoma	tumor cells and stroma		[64]
	prostate cancer	tumor cells	expression influenced by differentiation	[66]
αvβ6	basal cell carcinoma	tumor cells	higher expression frequency in infiltrative subtype	[67]
	breast cancer		expression correlates with prognosis	[68]
	colon cancer		upregulated at invasive front and in budding tumor cells	[69]
	endometrial cancer		often overexpressed without correlation with occurrence of lymph node metastasis	[70]
	gastric cancer		potential prognostic marker in early stage carcinoma	[53]
	liver		differentiates cholangiocarcinoma from hepatocellular carcinoma	[52]
	non small cell lung cancer		high expression frequency with intratumoral heterogeneity, no correlation with survival	[71]
	lung cancer brain metastases		high expression frequency	[62]
	oral squamous cell carcinoma		expression at invasive front	[72]
	ovarian cancer		expression correlates with grade	[55]
	pancreatic cancer		high expression frequency	[51] [73]
	prostate cancer		not/weakly expressed	[66]
αvβ8	non small cell lung cancer	tumor cells	low to moderate expression frequency, no correlation with survival	[63]
	prostate cancer		not expressed	[66]
α5β1	oral squamous cell carcinoma	tumor, endothelial cells, stroma	strong expression in stroma, expressed also in tumor and endothelial cells	[64]
	ovarian cancer	tumor cells	moderate expression frequency, correlates with survival	[74]

## Abbreviations

RGD	Arg-Gly-Asp
BM	Basement membrane
BMP	Bone morphogenetic protein
CAM	Cell adhesion molecule
CD	Crohn’s disease
COPD	Chronic obstructive pulmonary disease
DC	Dendritic cells
ECM	Extracellular matrix
ERK	Extracellular signal regulated kinase
FA	Focal adhesion
TGF-β	Transforming growth factors-β
MAP	Mitogen-activated protein kinases
PI3K	Phosphoinositide kinase
GTP	Guanosine-5′-triphosphate
EGF-R	Epidermal growth factor receptor
VEGF	Vascular endothelial growth factor
PDGF-R	Platelet-derived growth factor
FAK	Focal adhesion kinase
CAS	Crk-associated substrate
LAP	Latency-associated peptide
MMP	Matrix metalloproteases
uPA	Urokinase-type plasminogen activator
HREs	Hypoxia response elements
HAX-1	Hematopoietic lineage cell-specific protein-1 (HS1) associated protein
HIF	Hypoxia-inducible factor
OSCC	Oral squamous cell carcinoma
BCC	Basal cell carcinomas
Shh	Sonic hedgehog
SPECT	Single-photon emission computed tomography
tTregs	T regulatory cells
SLC	Small latent complex
LLC	Large latent complex
LTBP	Latent TGF- β binding proteins
FMDV	Foot-and-mouth disease virus
HNSCC	Head and neck squamous cell carcinoma
NSCLC	Non-small-cell lung carcinoma
PDAC	Pancreatic carcinoma
SPECT	Single photon emission computed tomography
PET	Positron emission tomography
